# Chemical Purification of Terbium-155 from Pseudo-Isobaric Impurities in a Mass Separated Source Produced at CERN

**DOI:** 10.1038/s41598-019-47463-3

**Published:** 2019-07-26

**Authors:** Ben Webster, Peter Ivanov, Ben Russell, Sean Collins, Thierry Stora, Joao Pedro Ramos, Ulli Köster, Andrew Paul Robinson, David Read

**Affiliations:** 1 National Physical Laboratory, Teddington, TW11 0LW UK; 2 Department of Chemistry, University of Surrey, Guildford, GU2 7XH UK; 3 CERN - European Organization for Nuclear Research, Esplanade des Particules 1, 1217 Meyrin, Switzerland; 4 KU Leuven, Institute for Nuclear and Radiation Physics, Celestijnenlaan 200D, 3001 Heverlee, Belgium; 5 Institut Laue-Langevin, 38042 Grenoble, France; 6 Christie Medical Physics and Engineering (CMPE), The Christie NHS Foundation Trust, Manchester, M20 4BX UK; 7 University of Manchester, Manchester, M13 9PL UK

**Keywords:** Nuclear chemistry, Analytical chemistry

## Abstract

Four terbium radioisotopes (^149, 152, 155, 161^Tb) constitute a potential theranostic quartet for cancer treatment but require any derived radiopharmaceutical to be essentially free of impurities. Terbium-155 prepared by proton irradiation and on-line mass separation at the CERN-ISOLDE and CERN-MEDICIS facilities contains radioactive ^139^Ce^16^O and also zinc or gold, depending on the catcher foil used. A method using ion-exchange and extraction chromatography resins in two column separation steps has been developed to isolate ^155^Tb with a chemical yield of ≥95% and radionuclidic purity ≥99.9%. Conversion of terbium into a form suitable for chelation to targeting molecules in diagnostic nuclear medicine is presented. The resulting ^155^Tb preparations are suitable for the determination of absolute activity, SPECT phantom imaging studies and pre-clinical trials.

## Introduction

Four terbium isotopes (^149,152,155,161^Tb) have been identified as having suitable physical properties (i.e. half-life (*T*_1/2_); emission type and quantity of emitted radiation) for use in cancer treatment and diagnosis. (Table 1)^1–3^. Initial pre-clinical trials^1^ have highlighted all four isotopes as being theranostic candidates using a folate-receptor derivative, cm09. Terbium isotopes form stable complexes with DOTA-containing targeting agents which show favourable *in-vivo* stability, emphasising their suitability for clinical use^1,4,5^. Terbium-155 (*T*_*1/2*_ = 5.32 d^3^) offers promise as an imaging tracer in single photon emission computed tomography (SPECT), with initial pre-clinical studies indicating excellent image quality even at low doses^4^. The administration of ^155^Tb alongside a therapeutic terbium isotope would give a theranostic pair with identical chemical properties; this is particularly advantageous as it facilitates the application of personalised medicine.

**Table 1:** Physical properties of four terbium isotopes and their applications in nuclear medicine^1,2^.

Isotope	T_1/2_	Decay mode (branching ratio)	Energy of particle radiation	Energy of main γ and X-ray emissions (keV)	Application			
					α therapy	PET	SPECT	β/auger therapy
^149^Tb	4.12 h	α (16.7%) β^+^ (7.1%)	E_α_ = 3.967 MeV E_β+mean_ = 730 keV	352 (29%) 165 (26%)	x	x		
^152^Tb	17.5 h	β^+^ (17%)	E_β+mean_ = 1.080 MeV	344 (64%)		x		
^155^Tb	5.32 d	EC (100%)	—	43 (86%) 49 (20%) 87 (32%) 105 (25%)			x	
^161^Tb	6.89 d	β^−^ (100%)	E_β-mean_ = 154 keV	26 (23%) 45–46 (18%) 49 (17%) 75 (10%)			x	x

EC – electron capture; PET – positron emission tomography; SPECT – single photon emission computed tomography.

Terbium-161 can be generated via neutron activation of a ^160^Gd target and subsequent decay of the ^161^Gd product to give the desired ^161^Tb (^160^Gd(n,γ)^161^Gd (β^−^) ^161^Tb)^5^. The other isotopes (^149,152,155^Tb) have been produced mainly via a proton-induced spallation reaction on a tantalum target combined with on-line mass separation at the CERN-ISOLDE facility^6–8^. A high percentage of the 1.4 GeV protons delivered by the proton synchrotron booster do not interact with the ISOLDE targets and therefore, the CERN-MEDICIS facility was established to produce isotopes for medical applications by inducing spallation reactions in a secondary target. At CERN-MEDICIS, off-line mass separation is applied to isolate isotopes of the same A/q value^6,7^. Terbium-155 sources used in this study were collected after mass-separation by implantation of the ion beam (155 A/q) into zinc-coated gold foils. Further chemical separation is still required as mass separation is unable to differentiate between isobaric and pseudo-isobaric species. Removal of the foil matrix is also required.

Alternative methods of producing these isotopes have been investigated (Table 2)^8–15^. However, full-scale production at a radionuclide purity sufficient for clinical studies has not yet been demonstrated.

**Table 2:** Established and alternative production methods for the four terbium isotopes.

Isotope	Nuclear reactions	Production facility	Incident particle energy	References
^149^Tb	Ta(p,sp)^149^Tb	Synchrotron	1.4 GeV (CERN)	Allen *et al*.^8^
	^151^Eu(^3^He, 5n)^149^Tb	Cyclotron	40–70 MeV	Zagryadskii *et al*.^14^
^152^Tb	Ta(p,sp)^152^Tb	Synchrotron	1.4 GeV (CERN)	Allen *et al*.^8^
	^155^Gd(p,4n)^152^Tb	Cyclotron	39 MeV	Steyn *et al*.^9^
^155^Tb	Ta(p,sp)^155^Tb	Synchrotron	1.4 GeV (CERN)	Allen *et al*.^8^
	^155^Gd(p,n)^155^Tb	Cyclotron	11 MeV	Vermeulen *et al*.^13^
	^153^Eu(ɑ,n)^155^Tb	Cyclotron	28 MeV	Kazakov *et al*.^12^
^161^Tb	^160^Gd(n,γ)^161^Gd - > ^161^Tb	Nuclear reactor	(flux = 8 × 10^14^ neutrons cm^−2^ s^−1^)	Lehenberger *et al*.^5^

All lanthanides, especially neighbouring elements, have similar chemical properties due to small differences in their ionic size/charge ratio, making the isolation of high purity individual lanthanide solutions challenging^16^. They exist predominately in the III+ oxidation state under aqueous conditions. The exceptions are europium, which can be selectively reduced to Eu(II) under strongly reducing conditions, and cerium, which can be easily oxidised to Ce(IV). Changes in oxidation state markedly influence chromatographic behaviour and this can be exploited when developing separation methods.

A well-known method of separating lanthanide elements utilises cation-exchange chromatography with α-hydroxyisobutyric acid (α-HIBA) eluent and provides good separation even from neighbouring elements^1,5,8^. However, the process is slow and requires precise control of chemical conditions (pH and α-HIBA concentration) to give optimal yield and purity. Attempts to accelerate separation tend to compromise terbium recovery.

A significant ^139^Ce (*T*_*1/2*_ = 137.6 d^17^) impurity exists in ^155^Tb sources from CERN-ISOLDE and CERN-MEDICIS owing to formation of the pseudo-isobaric species, ^139^Ce^16^O, which cannot be removed by mass separation. Given its half-life, it constitutes an increasing proportion of overall source activity during transport and storage. In this study, we present a simple method for producing radiologically pure terbium preparations in a chemical form suitable for chelation to targeting molecules as well as for absolute activity measurements and phantom imaging studies. Our aim was to develop a robust, efficient and rapid method capable of isolating terbium from the foil matrix as well as from ^139^Ce by selective oxidation. Therefore, ion-exchange and extraction chromatography resins were chosen based on their selectivity for tetravalent over trivalent species.

## Results

### Chemical separation

#### Batch separation

**Figure 1:**
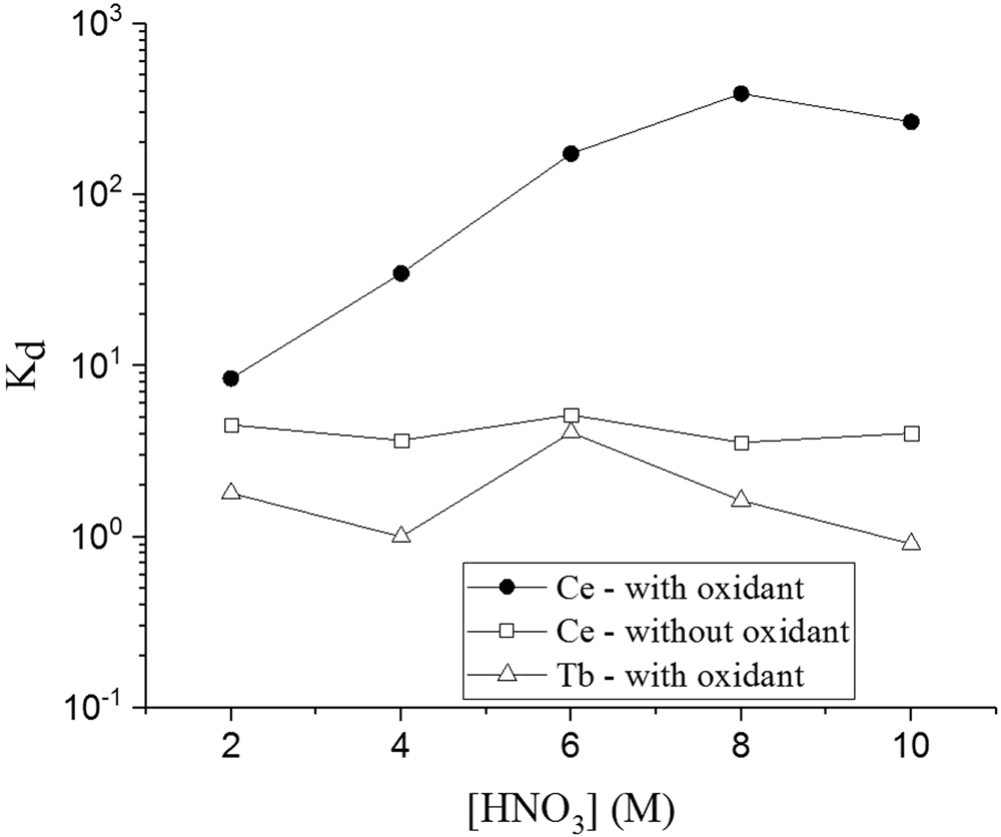
Distribution coefficients (*K*_*d*_) for Ce(IV), Ce(III) and Tb(III) in HNO_3_ solutions on UTEVA extraction chromatography resin. (N = 3).

In the presence of an oxidant (sodium bromate, NaBrO_3_) and in HNO_3_ solutions commercial UTEVA, TEVA and TK100 extraction resins (*Triskem International*) and AG1 anion exchange resin (*BioRad*) all showed pronounced cerium adsorption selectivity over terbium (Fig. 1). The results imply oxidation of cerium to Ce(IV) was achieved, with terbium remaining in the trivalent state (Tb(III)).

**Figure 2:**
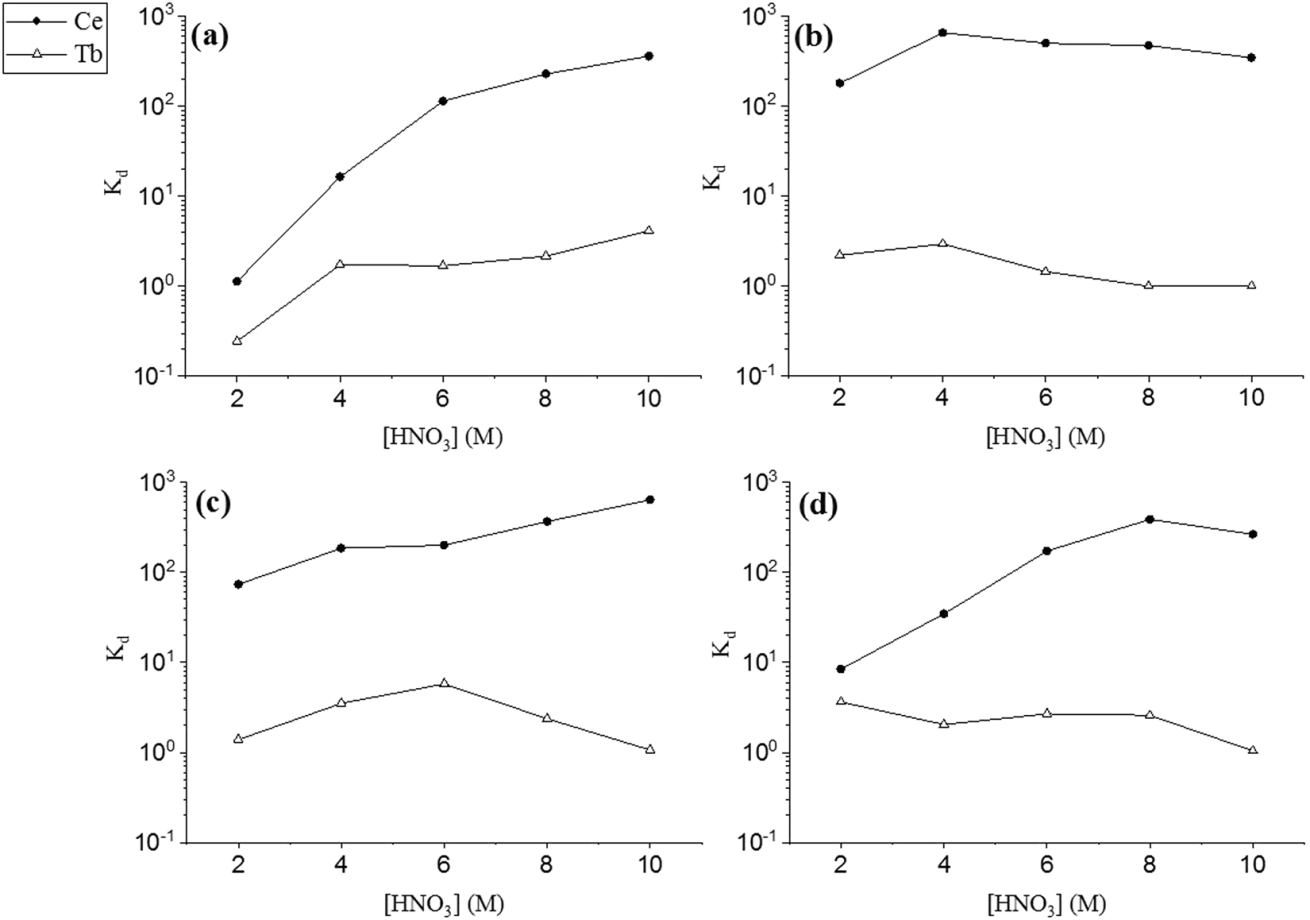
Distribution coefficients (K_d_) of Ce(IV) and Tb(III) in HNO_3_ solutions on (**a**) AG1 ion exchange resin, (**b**) TEVA resin, (**c**) TK100 resin, (**d**) UTEVA resin. (N = 3).

High Ce adsorption (*K*_*d*_ = 100–1,000) was observed at high HNO_3_ concentrations (8–10 M) on all four resins, whilst terbium adsorption remained minimal (*K*_*d*_ = 0.1–10) across the concentration range (Fig. 2). The best separation resolutions (Equation (2), SR > 100) were obtained using TEVA and UTEVA resins at high HNO_3_ concentrations; further studies were conducted on these resins using pre-packed cartridges.

#### Kinetic studies

**Figure 3:**
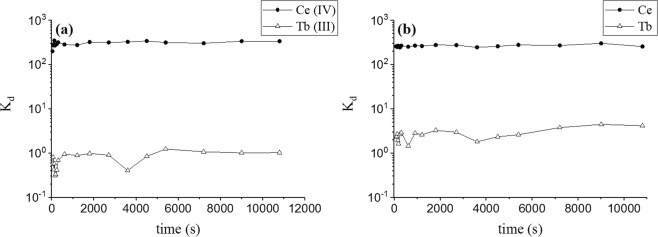
Kinetics of (**a**) the adsorption of Ce(IV) and Tb(III) onto UTEVA resin, and (**b**) the oxidation of cerium using sodium bromate. Measured as the distribution coefficient (K_d_) as a function of time.

UTEVA extraction chromatography resin was chosen to demonstrate kinetic behaviour with the rate of cerium adsorption studied in 10 M HNO_3_/0.1 M NaBrO_3_ solutions; rapid adsorption (<60 s) was observed (Fig. 3a). The rate of cerium oxidation was also studied in 10 M HNO_3_/0.1 M NaBrO_3_ solutions. Solutions were filtered under vacuum after a minimum of 60 s in contact with the resin. Rapid oxidation (<90 s) of cerium was observed (Fig. 3b).

Neither the rate of adsorption nor the rate of oxidation were limiting factors in the separation, suggesting that rapid column separation is achievable under these conditions.

#### Column studies

**Figure 4:**
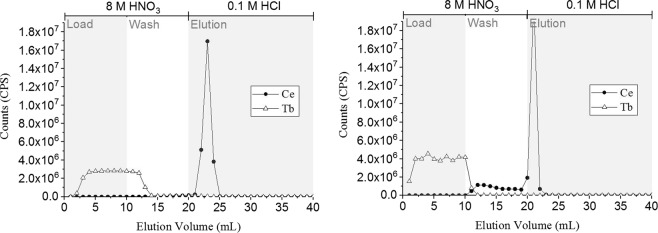
Elution profiles (N = 3) for Tb and Ce from a pre-conditioned 2 mL UTEVA cartridge (left) and a pre-conditioned 2 mL TEVA cartridge (right). Approximate flow rate = 0.3 mL/min.

Column-based separation using a commercially available pre-packed UTEVA cartridge (2 mL) provided effective isolation of terbium from cerium impurities. The elution profile (Fig. 4) shows that terbium (>99%) was removed in the load solution (10 mL, 8 M HNO_3_) and the subsequent wash solution (10 mL, 8 M HNO_3_) with minimal cerium impurities remaining (<0.002%). Cerium was successfully recovered by elution from the cartridge in hydrochloric solution (<10 mL, 0.1 M). The column-based separation was repeated using a pre-packed TEVA cartridge (2 mL); however, the separation achieved was less successful as ~0.1% Ce was detected in the Tb fraction under similar conditions (Fig. 4).

**Figure 5:**
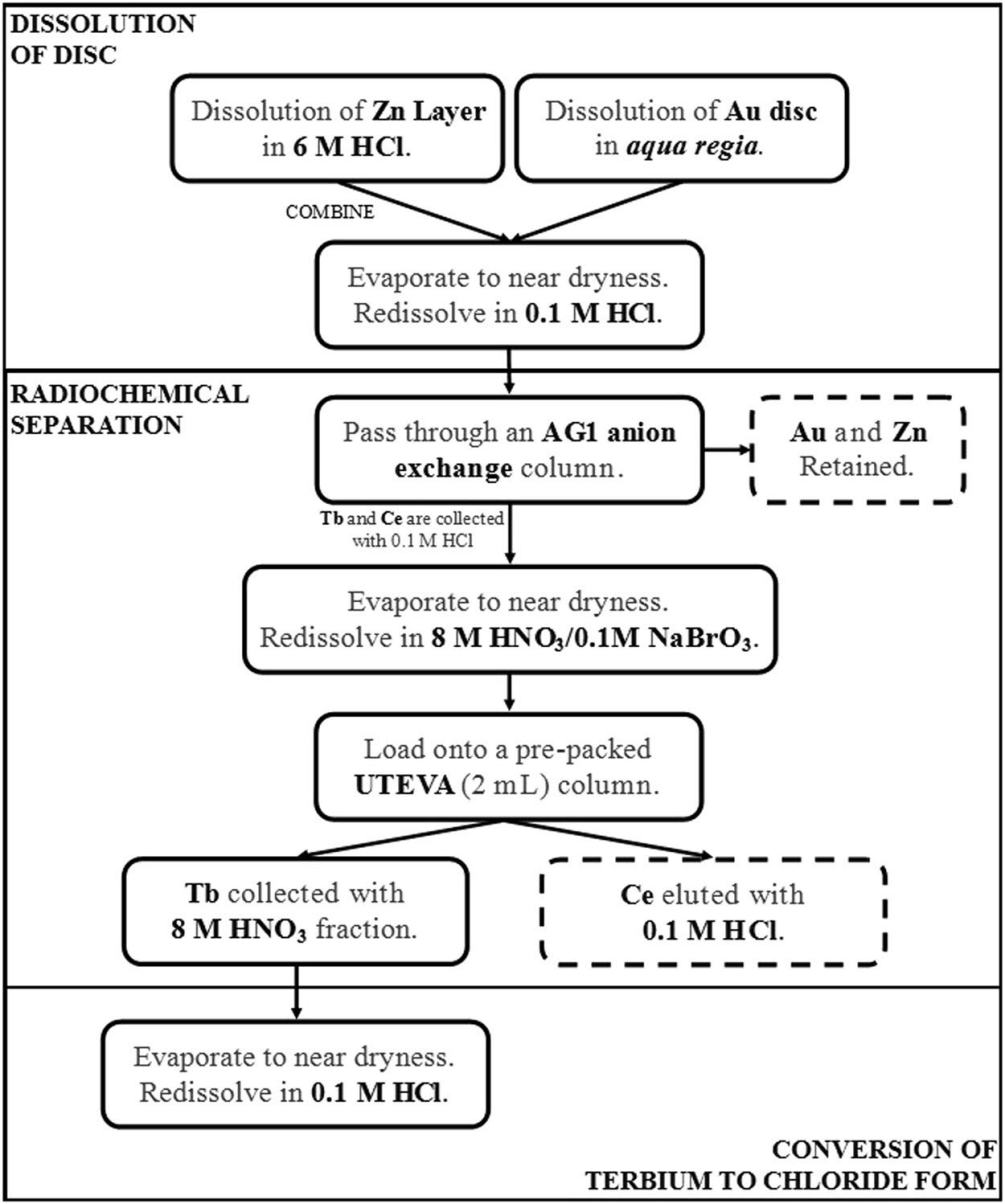
Final ^155^Tb separation scheme for CERN-ISOLDE and CERN-MEDICIS sources with an additional ^139^Ce recovery step.

These column studies allowed the development of a separation scheme for the removal of ^155^Tb from both ^139^Ce isotopic impurities and the zinc-plated gold catcher foil matrix (Fig. 5).

#### Validation with active ^155^Tb

The method has been validated on three sources (nominal ^155^Tb activity Bq – MBq) over a two year period. A mass separated source received from CERN-ISOLDE in 2017 (EOB - 07/09/2017 18:42:00) contained a significant ^139^Ce impurity (*A*_0_(^139^Ce)/*A*_0_(^155^Tb) = 0.30 ± 0.02), Table 3). The scheme detailed in Fig. 5 removed ^139^Ce to the level of the Compton continuum background (*D*_*L*,0_(^139^Ce)/*A*_0_(^155^Tb) = 0.00021). Total terbium fraction recovery, R_0,1_(^155^Tb)/R_0,2_(^155^Tb), was 0.973+/− 0.038 with a radiochemical purity of 99.98% (Table 3). This was consistent for all validation experiments.

**Table 3:** Radioisotopic composition of a ^155^Tb source received from CERN-ISOLDE before and after chemical separation (reference time 2017-09-29 12:00 UTC).

Isotope	*T*_*1/2*_	Activity of material supplied (MBq)	Activity following separation
^139^Ce	136.7 d	2.79 ± 0.068	≤1.90 kBq
^155^Tb	5.32 d	9.28 ± 0.63	9.03 ± 0.049 MBq

## Discussion

In many cases, it is essential that suitable radiochemical methods are available to provide radionuclides in sufficient quantities with relatively high specific activity, radionuclidic and chemical purity to facilitate accurate pre-clinical and clinical study. The method described is able to produce high radiological purity ^155^Tb sources, suitable for absolute activity, nuclear data and ionisation chamber measurements. The sources are also suitable for bioconjugation, molecular chelation and SPECT imaging studies. Although the ^139^Ce impurity discussed here does not possess significant biological toxicity^18^, it is radioactive and, if not removed, would result in an unnecessary additional dose to the patient.

Currently, proton-induced spallation is the main route for producing ^155^Tb at CERN for (pre)-clinical studies. The chemical purification method proposed here (Fig. 5) allows the quantitative separation of ^155^Tb from a zinc and/or gold matrix and from ^139^Ce impurities produced by spallation at the CERN-ISOLDE and CERN-MEDICIS facilities. The method is rapid, simple and can also be used to recover a high purity ^139^Ce source; a useful standard in gamma spectrometry (E_γ_ = 165.86 keV, 79.90%)^17^.

The method has not yet been validated for the removal of other stable (e.g. ^139^La^16^O^+^, ^155^Gd^+^) or longer-lived, radioactive (e.g. ^155^Eu^+^) isobaric impurities; as with ^139^Ce^16^O, they would not be removed by mass separation. Such impurities might not pose a significant toxicological risk if they were to enter the body^19,20^ but nevertheless, would form stable complexes with DOTA (logK > 22)^21^ and DOTA-containing targeting molecules^4^ and could compete with the target terbium isotope(s), reducing their efficacy.

## Materials and Methods

### Chemicals

Standard element solutions at starting concentrations of 1000 ppm were purchased from Johnson Matthey and Fluka Analytical (Tb and Ce, respectively). Mixed standard solutions were prepared in HNO_3_ (Trace Analysis Grade, Fisher Scientific) and diluted to the required concentration with ultrapure water (ELGA PURELAB Flex, Veolia Water, Marlow, UK, 18 MΩ cm, <5 ppb Total Organic Carbon). Anion exchange resin (Bio Rad AG1-X8, 100–200 mesh) and extraction chromatography resins (TEVA, TK100 and UTEVA, Triskem International 100–150 μm) were used throughout.

The ^155^Tb source was provided by CERN-ISOLDE and CERN-MEDICIS in the form of a zinc-coated gold foil. HCl (Trace Analysis Grade, Fisher Scientific) and HNO_3_ were used for foil dissolution and NaBrO_3_ (Alfa Aesar) for cerium oxidation.

### Inductively coupled plasma mass spectrometry (ICP-MS)

Measurement of stable ^140^Ce and ^159^Tb was carried out using a tandem ICP-MS/MS (Agilent 8800) equipped with a collision-reaction cell positioned between two quadrupole mass filters. The instument was run in Single Quad mode, with only the second mass filter operating. The instrument is fitted with a quartz double-pass spray chamber and a MicroMist nebuliser (Glass Expansion, Melbourne, Australia) and nickel sample and skimmer cones (Crawford Scientific, South Lanarkshire, UK). It was tuned daily using a mixed 1 ppb standard solution (Ce, Co, Li, Mg, Tl and Y in 2% v/v HNO_3_). No additional terbium-specific tuning was carried out. A ^209^Bi (10 ppb solution in 2% v/v HNO_3_) internal standard was used to monitor and correct for instrumental drift during longer runs. Blank HNO_3_ (2% v/v) solutions were monitored regularly to ensure no Ce or Tb cross-contamination during a run.

### Gamma-ray spectrometry

An n-type HPGe γ-ray spectrometer with a resolution (FWHM) of 1.79 keV at 1.33 MeV and relative efficiency 28% was used to determine the ^139^Ce/^155^Tb activity ratio. The detection system set-up and full-energy peak efficiency calibration is described in detail by Collins *et al*.^22^.

The nuclear data (half-lives and γ-ray emission intensities) used to determine the activities of ^155^Tb and ^139^Ce were taken from the evaluated database of ENSDF and the DDEP, respectively^3,17^. As ^139^Ce could not be observed after the chemical separation, the activity ratio of the ^139^Ce/^155^Tb in the chemically separated solution was estimated from the detection limit of the detector for ^139^Ce^23^.

### Irradiation conditions and mass separation

**Figure 6:**
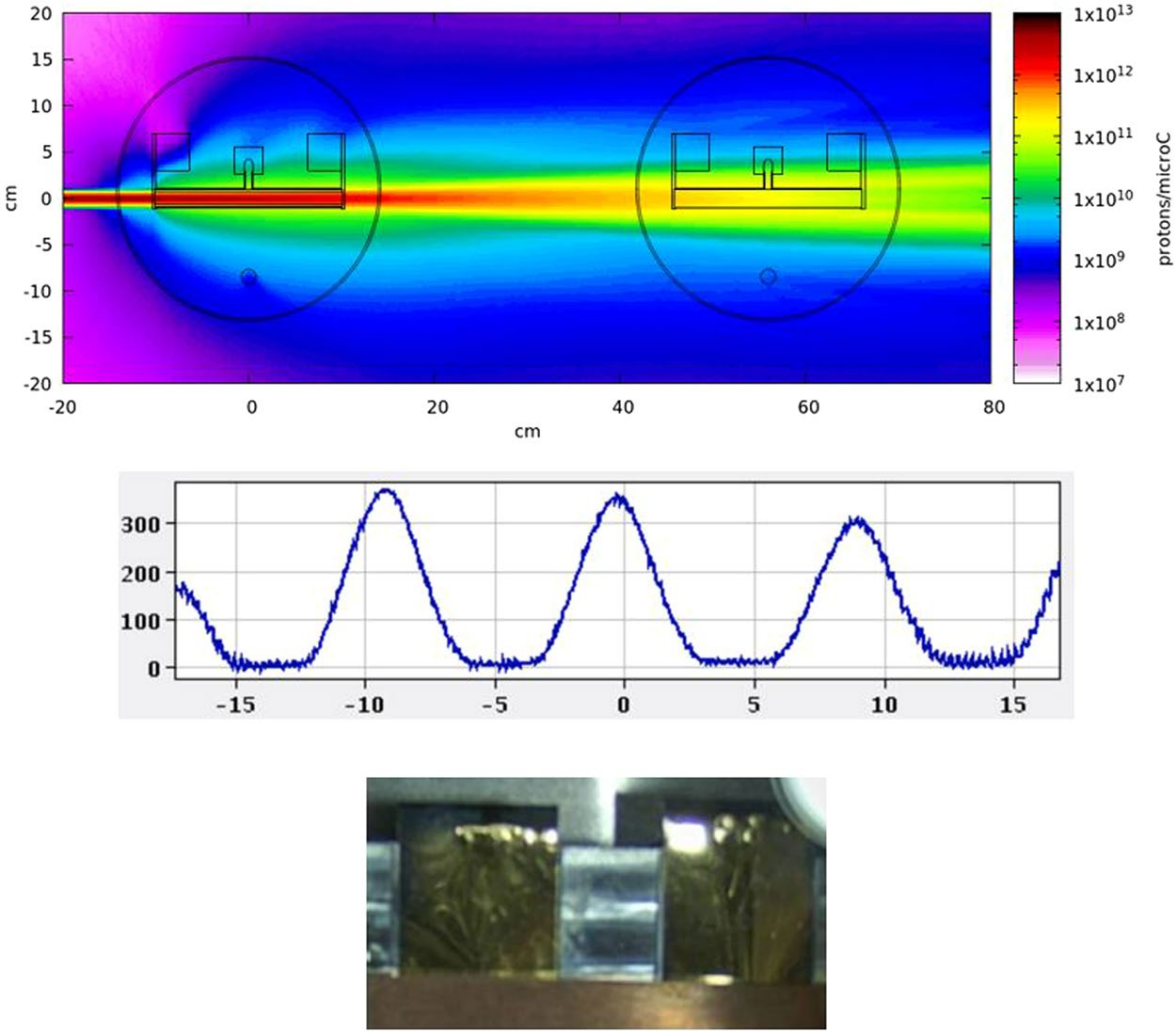
Top: Fluka simulation^25,26^ showing the incoming proton beam on an ISOLDE target (3.5 g/cm^2^ UC_x_ for the purpose of the simulation) and intercepting the MEDICIS target downstream. Middle: Screenshot taken with the beam scanner, located before the implantation chamber. Beams at A/q = 154,155,156 are seen (153, 157 partly visible). The collected beam is centred on A/q = 155, while isotopes present at other masses are physically removed from the implantation using mechanical slits located ahead of the foil. The horizontal scale is in mm. Bottom: Two zinc-coated gold foils in the collection chamber seen from the rear. The collection takes place on the foil located on the left.

Terbium-155 sources used in this study were produced at the CERN-ISOLDE and CERN-MEDICIS facilities. Three ^155^Tb sources were produced and provided to NPL for chemical separation between 2017 and 2018. The irradiation conducted at CERN-MEDICIS was as follows:

A high purity Ta metal target (*Ta647M*) made of 12 rolls of Ta foil (99.95% purity, 12 μm thick, 15 mm wide, 2 cm diameter) with a total mass of 357 g was arranged in a 20 cm long Ta tube coupled to a rhenium surface ion source. The target was irradiated with 1.4 GeV protons delivered by the Proton Synchrotron Booster accelerator (CERN, Geneva). The CERN-MEDICIS irradiation target is located in the High Resolution Separator (HRS) beam dump position at ISOLDE (Fig. 6), and receives a fraction of the scattered 1.8 × 10^18^ protons downstream from a primary HRS target (*623SiC*, ISOLDE physics program). The irradiation was scheduled within the MED004 approved experiment and took place from 27^th^ September to 1^st^ October 2018. The irradiated target was then moved to the CERN-MEDICIS isotope mass separator in order to release and extract ion species selected at mass-to-charge ratio of 155^6^. The separated ions were collected on a zinc-plated gold foil and removed on 3^rd^ October. The following isotopes were implanted upon sample retrieval: ^139^Ce (implanted as ^139^Ce^16^O^+^): 6.9 MBq; ^155^Dy: 3.6 MBq; ^155^Tb: 20 MBq. Upon reception at NPL, ^155^Dy had decayed below the detection limit (*D*_*L*,0_ (^155^Dy) = 1.75 kBq).

### Chemical separation

#### Batch separation studies

The adsorption of Tb and Ce onto ion exchange (AG1, BioRad) and extraction chromatography resins (TEVA, UTEVA and TK100, Triskem International) was studied over a range of HNO_3_ solution concentrations (2–10 M). Nitric acid solutions (2 mL) containing a mixture of 100 ppb stable Ce and Tb were prepared. An aliquot was taken from each solution for ICP-MS measurement. The remaining solution was added to 0.1 g of resin (UTEVA, TEVA, TK100 or AG1). Sodium bromate (0.1 M, 0.03 g) was added to identical samples to assess changes in adsorption to the resin as a result of selective oxidation of Ce. In all cases, the samples were shaken and left to equilibrate for 24 h. After equilibration, the solutions were filtered to isolate the aqueous phase (Whatman 41 ashless filter paper, 20–25 μm pore size). An aliquot was taken from each sample, diluted with 2% HNO_3_ (2% v/v) and analysed by ICP-MS.

The adsorption of Tb and Ce onto each resin was quantified by calculating the distribution coefficient (*K*_*d*_) using Eq. (1)^24^.

1 $${k}_{d}=(\frac{{(CPS)}_{0}-{(CPS)}_{t}}{{(CPS)}_{t}})\times (\frac{V}{m})$$

Where *(CPS)*_0_ and *(CPS)*_*t*_ are the concentrations of analyte in the aqueous phase before and after equilibration, respectively, *V* is the volume of solution (mL) and *m* is the mass of resin used (g).

The separation achievable in the different HNO_3_ solutions was quantified by calculating the separation factor using Eq. (2).

2 $${\rm{SF}}=(\frac{{k}_{d}(Tb)}{{k}_{d}(Ce)})$$

#### Kinetic studies

In order to determine the rate at which Ce(IV) and Tb(III) are adsorbed onto UTEVA, a HNO_3_ (10 M) solution containing 100 ppb Ce, 100 ppb Tb and sodium bromate (0.1 M) was left for 24 h to allow for the oxidation of Ce(III) to Ce(IV). Aliquots (2 mL) were added to vials containing UTEVA resin (0.1 g) and were left in static conditions before being filtered to isolate the aqueous phase at regular time intervals under vacuum (60 seconds–180 minutes).

Likewise, to determine the rate at which Ce is oxidised, an excess of sodium bromate (0.1 M, 0.03 g) was added to a HNO_3_ solution (2 mL, 10 M) containing 100 ppb Ce, 100 ppb Tb and 0.1 g of UTEVA resin. Repeat samples were left in static conditions before being filtered to isolate the aqueous phase at regular time intervals under vacuum (90 seconds - 180 minutes).

An aliquot of each filtrate was diluted with HNO_3_ (2% v/v) before analysis by ICP-MS. Distribution coefficients were calculated using Eq. (1).

#### Column studies

Column-based separation was studied using a pre-packed 2 mL UTEVA cartridge (50–100 µm, *Triskem International*). The resin was pre-conditioned with 8 M HNO_3_ (20 mL). A HNO_3_ solution (10 mL, 8 M) containing 0.1 M NaBrO_3_, 100 ppb Tb and 100 ppb Ce was loaded onto the resin. A wash solution of 10 mL 8 M HNO_3_ was added to ensure removal of all Tb from the cartridge. Subsequent elution of Ce was achieved using 20 mL 0.1 M HCl. This separation method was also repeated using a pre-packed 2 mL TEVA cartridge (50–100 µm, *Triskem International*).

Throughout the separations, 1 mL fractions were collected, diluted with HNO_3_ (2% v/v) and analysed by ICP-MS in order to compile an elution profile. Column separations were carried out under gravity (approximate flow rate = 0.3 mL/min).

### Method validation with active sample

Three zinc-coated gold foils containing ^155^Tb and ^139^Ce were received at NPL from CERN-ISOLDE and CERN-MEDICIS. The radionuclides were leached by dissolving the zinc layer in 20 mL 6 M HCl and the gold foil in 20 mL *aqua regia*. Both layers were dissolved in order to maximise the yield of terbium from the sources received. The combined solution was evaporated gently on a hot plate (~150 °C) to incipient dryness and re-dissolved in a 10 mL 8 M HNO_3_/0.1 M NaBrO_3_ solution. An ampoule was prepared for HPGe gamma spectrometry in order to quantify the activity of ^155^Tb and ^139^Ce present. After analysis, the portion was recombined with the bulk solution.

A pre-packed 2 mL UTEVA cartridge (*Triskem International*, 50–100 µm) was conditioned with 20 mL 8 M HNO_3_. The 10 mL sample was then loaded onto the column and the fraction collected under gravity. The column was washed with 10 mL 8 M HNO_3_. This fraction was collected, under gravity, and combined with the load fraction. The combined fractions were evaporated gently on a hot plate (~150 °C) to incipient dryness and re-dissolved in 20 mL 0.1 M HCl.

An ampoule of the combined terbium fractions was prepared and analysed by HPGe gamma spectrometry in order to assess the resultant purity of the ^155^Tb source after separation. The terbium recovery was calculated as follows:

3 $$\frac{{R}_{0,2}}{{R}_{0,1}}=\frac{\frac{{N}_{2}}{{\rm{\Delta }}{{t}}_{L,2}\cdot {m}_{2}}\cdot {e}^{-\lambda {\rm{\Delta }}{t}_{2}}\frac{{\rm{\Delta }}{{t}}_{{\rm{2}}}\cdot \lambda }{(1\cdot {e}^{-\lambda {\rm{\Delta }}{t}_{2}})}\cdot {m}_{E}}{\frac{{N}_{1}}{{\rm{\Delta }}{{t}}_{L,1}\cdot {m}_{1}}\cdot {e}^{-\lambda {\rm{\Delta }}{t}_{1}}\frac{{\rm{\Delta }}{{t}}_{{\rm{1}}}\cdot \lambda }{(1\cdot {e}^{-\lambda {\rm{\Delta }}{t}_{1}})}\cdot {m}_{D}}$$

where *R*_0,1_ and *R*_0,2_ are the count rates of the 105 keV gamma-ray emission before and after separation of the ^139^Ce, respectively at the reference time 2017-09-29 12:00 UTC. *N*_1_ and *N*_2_ are the net peak areas of the 105 keV full-energy peak measured before and after separation, Δt_L,1_ and Δt_L,1_ are the measurement live times, m_1_ and m_2_ are the measured active masses of solution, m_D_ and m_E_ are the total mass of solution used to dissolve the Zn layer of the target and eluent used in the chemical separation, respectively, λ is the decay constant of ^155^Tb, t_1_ and t_2_ are the time elapsed since the reference time and Δt_1_ and Δt_2_ are the measurement real times.

## Conclusion

A novel method has been developed for the isolation of ^155^Tb from sources produced at CERN-ISOLDE and CERN-MEDICIS, currently the main producers of the isotope. A high purity ^155^Tb preparation was successfully recovered from a zinc-coated gold matrix and from ^139^Ce impurities using a chromatography-based system. The method was shown to be capable of separating 100 ppb Tb and Ce in a 10 mL solution, equivalent to ~6 GBq ^155^Tb and ~0.25 GBq ^139^Ce. The radiologically pure ^155^Tb preparation was subsequently used for absolute activity measurements and ion chamber measurements. The preparations are also suitable for phantom imaging and pre-clinical studies.

## Data Availability

The data generated and analysed during this study are available, upon reasonable request, from the corresponding author.
